# The Synthesis of the Metabolites of 2′,3′,5′-Tri-*O*-acetyl-*N*_6_-(3-hydroxyphenyl) Adenosine (WS070117)

**DOI:** 10.3390/molecules21010008

**Published:** 2015-12-28

**Authors:** Wen-Xuan Zhang, Hong-Na Wu, Bo Li, Hong-Lin Wu, Dong-Mei Wang, Song Wu

**Affiliations:** State Key Laboratory of Bioactive Substance and Function of Natural Medicines, Institute of Materia Medica, Peking Union Medical College and Chinese Academy of Medical Sciences, Beijing 100050, China; wxzhang@imm.ac.cn (W.-X.Z.); hongnawu@imm.ac.cn (H.-N.W.); bolee@imm.ac.cn (B.L.); redwoods86@sina.com (H.-L.W.); wangdmchina@imm.ac.cn (D.-M.W.)

**Keywords:** WS070117, metabolites, synthesis

## Abstract

Seven metabolites of 2′,3′,5′-tri-*O*-acetyl-*N*_6_-(3-hydroxyphenyl) adenosine (WS070117) were synthesized by deacetylation, hydrolysis, cyclization, sulfonylation and glycosylation reactions, respectively. All these compounds, which could be useful as material standards for metabolic research, were characterized by NMR and HPLC-MS (ESI) analyses.

## 1. Introduction

**Figure 1:**
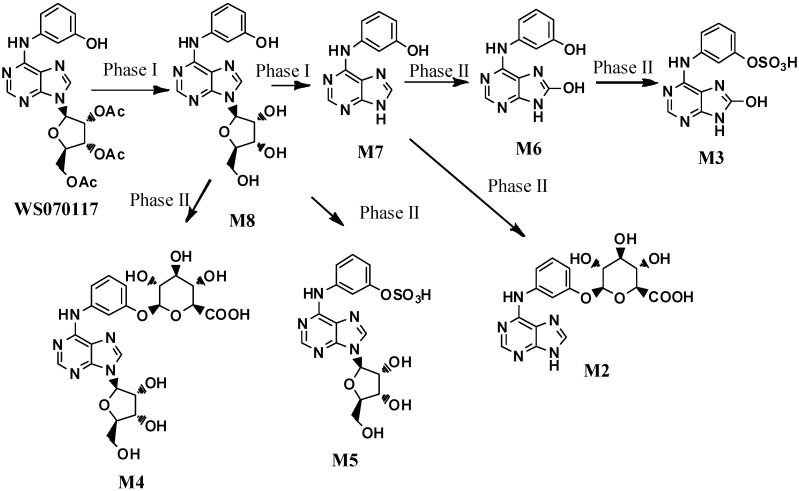
The structures of the WS070117 metabolites in rat urine.

2′,3′,5′-Tri-*O*-acetyl-*N*_6_-(3-hydroxyphenyl) adenosine (also known as WS070117) is a new adenosine analog anti-hyperlipidemic drug candidate currently in many preclinical studies [1,2,3,4,5]. Guo and coworkers have investigated and elucidated the *in vivo* metabolites of WS070117 in rat urine after oral administration of WS070117 by HPLC-DAD, ESI-MS and Off-Line Microprobe NMR [6]. In the study, seven metabolites of WS070117 were observed in the HPLC trace (the metabolites are ranked **M2**–**M8** according to the retention time; Figure 1). The structure elucidation results unambiguously revealed that there are two phase I metabolites, including a deacetylation product of WS070117 (**M8**) and an adenine derivative formed by the loss of ribofuranose (**M7**). In addition, there are five phase II metabolites: **M6** is the oxidation product of **M7** at C-8; **M2** and **M4** are the glycosylation products of **M7** and **M8** on the phenolic hydroxyl groups, respectively; **M3** and **M5** are the sulfonylation products of **M6** and **M8**, respectively. Herein, the synthesis of the above metabolites was carried out to provide metabolites material standards for preclinical pharmacokinetic studies.

## 2. Results and Discussion

**Scheme 1:**
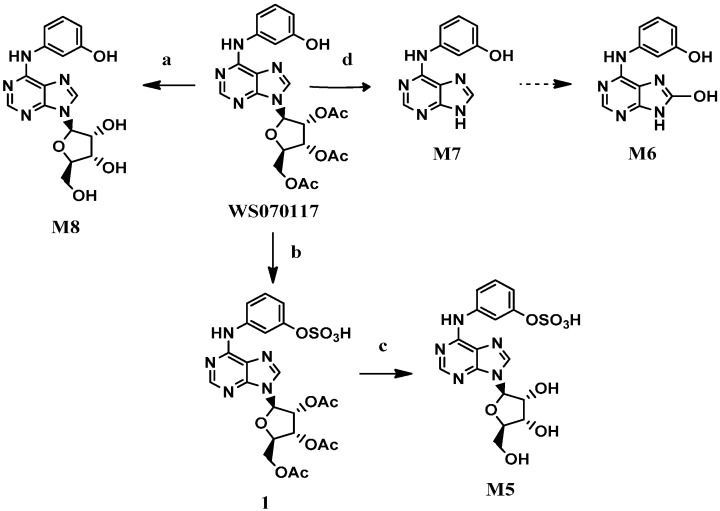
The synthesis of **M5**, **M7** and **M8**.

The metabolite **M8** was prepared in quantitative yield by the hydrolysis of the acetyl groups in WS070117 with NaOH [7] (Scheme 1). Treatment of WS070117 with chlorosulfonic acid [8] afforded the corresponding sulphonate **1**, which was converted to the desired product **M5** by subsequent deacetylation with Na_2_CO_3_. Direct hydrolysis of the glycosidic bond in WS070117 with concentrated hydrochloric acid and 95% ethanol solution at reflux smoothly afforded **M7**.

**Scheme 2:**
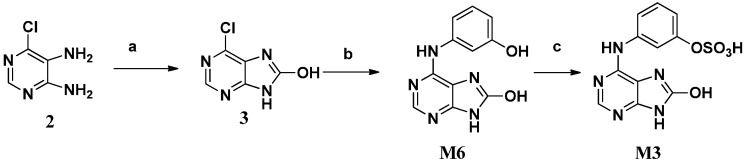
The synthesis of **M3** and **M6**.

We initially attempted to synthesize **M6** by bromination and hydrolysis reactions at C-8 of **M7** [9], but liquid bromine and NaH did not afford the needed C8-brominated adenine. Then 4,5-diamino-6-chloropyrimidine **2** and *N*,*N′*-carbonyldiimidazole (CDI) were employed to synthesize 8-hydroxy-6-chloroadenine (**3**) [10], which could be ammoniated at C-6 to prepare **M6** (Scheme 2). The amination was catalyzed by hydrochloric acid to afford the desired product in 70% yield at 120 °C in a microwave reactor. Subsequent sulfonylation of **M6** with sulfur trioxide-pyridine complex gave the target metabolite **M3**.

*Reagents and conditions*: (a) NaOH, H_2_O, 2 h, 99%; (b) HSO_3_Cl, pyridine, r.t., 12 h; (c) Na_2_CO_3_, H_2_O, 12 h, 66.3% for two steps; (d) HCl/95%EtOH, reflux,1 h, 92.5%.

*Reagents and conditions*: (a) CDI, dioxane, reflux, 6 h; (b) 3-aminophenol, HCl(aq), ethanol, MW 120 °C, 4 h, 70%; (c) SO_3_·pyr., pyridine, DMF, 24 h, 67.2%

**Scheme 3:**
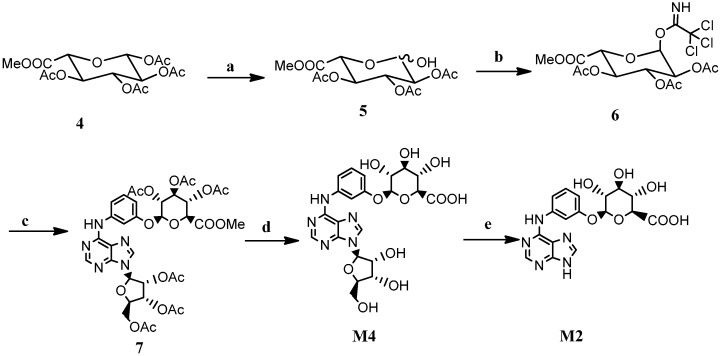
The synthesis of **M4** and **M2**.

Initially, the glycosylation reaction with tetra-*O*-acetyl-β-d-glucopyranuronic acid methyl ester as the donor and WS070117 as the receptor was catalyzed by SnCl_4_ to synthesize **M4**, but the yield of the desired product was poor, with the formation of a complex mixture of by-products. The Koenigs–Knorr glycosylation reaction with acetobromo-α-d-glucuronic acid methyl ester as the donor was then employed, but Ag_2_O failed to catalyze the reaction even at two equivalents, which may be due to its complexation with the nitrogen-atoms. Afterwards the Schmitt glycosylation reaction was tried with trichloroacetimidate as donor (Scheme 3). A series of conditions for selective anomeric deacetylation of **4** were investigated, including benzylamine/DMF [11], FeCl_3_·6H_2_O/CH_3_CN [12] and Nd(OTf)_3_/CH_3_OH [13]. The results showed that Nd(OTf)_3_/CH_3_OH gave a cleaner reaction in quantitative yield and the product could be used directly in the next step without purification. Under the catalysis of BF_3_·Et_2_O, trichloroacetimidate **6** reacted smoothly with WS070117 to give the desired β-glucoside **7** due the neighboring group participation effect. After deacetylation with Cs_2_CO_3_, **M4** was obtained in 83.3% yield. However, the glycosylation reaction of M7 under the same conditions only afforded the desired product **M2** with a 25% conversion because of the poor solubility of the raw material in dichloromethane. An alternate route such as selective hydrolysis of the N-glycoside bond of **M4** with aqueous hydrochloric acid solution afforded **M2** in moderate yield.

*Reagents and conditions*: (a) Nd(OTf)_3_/CH_3_OH, reflux, 6 h; (b) Cl_3_CCN/DBU/CH_2_Cl_2_, 0 °C, 2 h; (c) WS070117, BF_3_·Et_2_O, CH_2_Cl_2_, 0 °C,12 h, 86.7%; (d) Cs_2_CO_3_/MeOH/H_2_O, 40°C, 0.5 h, 83.3%; (e) HCl/H_2_O, reflux, 74.1%.

## 3. Materials and Methods

### 3.1. General Information

WS070117 was prepared in our laboratory in 98% purity. Unless otherwise indicated, all commercial reagents and solvents were used without additional purification. ^1^H-NMR and ^13^C-NMR spectra were recorded on a Mercury-300M (Varian, Salt Lake City, UT, USA,) or Bruker 400M (Varian) spectrometer. Chemical shifts (in ppm) were referenced to tetramethylsilane (δ = 0) in deuterated solvent as internal standard. HPLC-mass spectra (ESI) were recorded on an Acquity UPLC-MS system (Waters, Milford, MA, UK). Optical rotations were recorded on p-2000 polarimeter (Jasco, Tokyo, Japan) at 20 °C in 589 nm.

### 3.2. Synthesis of N_6_-(3-Hydroxyphenyl) Adenosine *(**M8**)*

NaOH (2.1 g, 52.5 mmol) was added to a stirred suspension of WS070117 (8.0 g, 16.5 mmol) in H_2_O (50 mL), and the mixture was stirred at room temperature for 2 h until the disappearance of the solid. The solution was cooled to 5 °C and the precipitated solid was filtered and washed with water to give **M8** (5.90 g, 99%) as a white solid, ${\left[\alpha \right]}_{D}^{20}$ = −40.7 (*c* = 0.4, H_2_O), ESI/MS: 360.15 [M + H]^+^, ^1^H-NMR (300 MHz, methanol-*d*_4_, see Supplementary Materials) δ 8.34 (d, *J* = 5.0 Hz, 2H), 7.27 (t, *J* = 2.1 Hz, 1H), 7.15–6.99 (m, 2H), 6.52 (dt, *J* = 7.5, 1.8 Hz, 1H), 6.00 (d, *J* = 6.3 Hz, 1H), 4.77 (dd, *J* = 6.3, 5.1 Hz, 1H), 4.34 (dd, *J* = 5.1, 2.6 Hz, 1H), 4.19 (q, *J* = 2.6 Hz, 1H), 3.90 (dd, *J* = 12.6, 2.6 Hz, 1H), 3.76 (dd, *J* = 12.5, 2.8 Hz, 1H). ^13^C-NMR (100 MHz, methanol-*d*_4_) δ 161.6, 156.5, 155.8, 152.5, 151.9, 144.8, 143.9, 133.1, 115.6, 114.4, 111.6, 93.9, 90.7, 78.1, 75.2, 66.0.

### 3.3. Synthesis of N_6_-(3-O-Sulfophenyl) Adenosine *(**M5**)*

Chlorosulfonic acid (300 μL, 4.6 mmol) was added dropwise (*carefully!*) into a stirred solution of WS070117 (130 mg, 0.27 mmol) in dry pyridine (3 mL) at 0 °C under N_2_. The solution was warmed to room temperature and stirred for 12 h. Na_2_CO_3_ (aq, 0.5 M, 20 mL) was then added to adjust the pH to 10. The reaction mixture was stirred for another 12 h at room temperature, and the solvent was removed *in vacuo* and the remaining solid was purified by Sephadex LH-20 chromatography eluting with water to give **M5** (78 mg, 66.3%) as a white solid, ${\left[\alpha \right]}_{D}^{20}$ = −33.5 (*c* = 0.15, H_2_O), ESI/MS: 440.10 [M + H]^+^, ^1^H-NMR (300 MHz, D_2_O, see Supplementary Materials) δ 8.28 (s, 1H), 8.21 (s, 1H), 7.50 (t, *J* = 3.8 Hz, 1H), 7.40 (t, *J* = 5.7 Hz, 2H), 7.10 (p, *J* = 7.7 Hz, 1H), 6.03 (dd, *J* = 5.9, 2.6 Hz, 1H), 4.76–4.67 (m, 1H), 4.41 (dd, *J* = 5.4, 3.7 Hz, 1H), 4.28 (dq, *J* = 6.2, 3.4 Hz, 1H), 3.93 (dd, *J* = 12.9, 2.94 Hz, 1H), 3.83 (dd, *J* = 12.9, 3.7 Hz, 1H). ^13^C-NMR (100 MHz, D_2_O) δ180.2, 152.0, 151.5, 148.4, 140.9, 138.6, 130.1, 120.1, 117.7, 115.4, 88.5, 85.8, 73.9, 70.7, 68.0, 61.5.

### 3.4. Synthesis of N_6_-(3-Hydroxyphenyl) Adenine *(**M7**)*

Concentrated hydrochloric acid (5 mL) was added into a stirred solution of WS070117 (4.85 g, 10 mmol) in 95% ethanol (100 mL). The reaction was refluxed for 1 h and then cooled to room temperature. The precipitated solid was filtered and washed with water to give **M7** (2.1 g, 92.5%) as a white solid, ESI/MS: 228.05 [M + H]^+^, ^1^H-NMR (300 MHz, DMSO-*d*_6_, see Supplementary Materials) δ11.22 (s, 1H), 9.61 (brs, 1H), 8.74 (s, 1H), 8.70 (s, 1H), 7.42 (s, 1H), 7.31 (d, *J* = 10.4 Hz, 1H), 7.20 (t, *J* = 8.0 Hz, 1H), 6.62 (d, *J* = 8.0 Hz, 1H). ^13^C-NMR (100 MHz, DMSO-*d*_6_) δ 158.3, 150.0, 148.9, 148.8, 143.8, 139.1, 130.1, 113.3, 112.6, 112.6, 109.0.

### 3.5. Synthesis of 8-Hydroxy-N_6_-(3-hydroxy-phenyl) Adenine *(**M6**)*

*N*,*N′*-Carbonyldiimidazole (CDI, 1.6 g, 10 mmol) was added into a stirred solution of 4,5-diamino-6-chloropyrimidine (**2**, 0.76 g, 5.23 mmol) in 1,4-dioxane. The reaction mixture was refluxed for 6 h and then cooled to room temperature. The precipitated solid was filtered and washed with water to give 8-hydroxy-6-chloroadenine (**3**, 700 mg). Concentrated hydrochloric acid (1 mL) was then added into a stirred solution of intermediate **3** (0.7 g, 4.12 mmol) and 3-aminophenol (0.7 g, 6.42 mmol) in ethanol (20 mL) in a sealed tube, which was irradiated in a microwave reactor at 120 °C for 4 h and then cooled to room temperature. The precipitating solid was filtered and washed with water to give **M6** (0.9g, 70%) as a white solid, ESI/MS: 244.07 [M + H]^+^, ^1^H-NMR (400 MHz, DMSO-*d*_6_, see Supplementary Materials) δ 11.69 (s, 1H), 10.80 (s, 1H), 9.43 (s, 1H), 8.25 (s, 1H), 7.33 (s, 1H), 7.22–7.10 (m, 2H), 6.48 (d, *J* = 4.0 Hz, 1H). ^13^C-NMR (100 MHz, DMSO-*d*_6_) δ 158.2, 152.9, 149.5, 148.1, 142.5, 140.9, 129.9, 110.8, 110.2, 107.1, 106.3.

### 3.6. Synthesis of 8-Hydroxy-N_6_-(3-O-sulfophenyl) Adenine *(**M3**)*

SO_3_·Pyr complex (9.54 g, 60 mmol) was added into a stirred solution of **M6** (2.27 g, 9.3 mmol) in pyridine (50 mL) and DMF (50 mL) at room temperature. After 24 h, acetone (200 mL) was added and the precipitated solid was filtered and washed with water to give **M3** (2.01 g, 67.2%) as a white solid, ^1^H-NMR (300 MHz, DMSO-*d*_6_) δ 8.84 (s), 8.20 (s, 1H), 7.61 (dd, *J* = 8.2, 2.3 Hz, 1H), 7.51 (dd, *J* = 2.3, 2.2 Hz, 1H), 7.18 (dd, *J* = 8.2 Hz, 1H), 6.77 (dd, *J* = 8.2, 2.2 Hz, 1H). ^13^C-NMR (100 MHz, DMSO-*d*_6_) δ 154.3, 153.5, 150.7, 149.4, 142.4, 141.2, 129.3, 114.5, 114.7, 111.4, 106.3.

### 3.7. Synthesis of Intermediate ***7***

Nd(OTf)_3_ (0.95 g, 1.6 mmol) was added into a stirred solution of tetra-*O*-acetyl-β-d-glucopyranuronic acid methyl ester (**4**, 6.0 g, 16 mmol) in methanol (200 mL). The solution was refluxed for 6 h and cooled to room temperature. The solution was then concentrated *in vacuo* and the residue was dissolved with CH_2_Cl_2_ (30 mL). The organic layer was washed with water, dried with Na_2_SO_4_ and concentrated *in vacuo* after filtration to give an orange oil of intermediate **5**. Next DBU (0.75 mL, 5 mmol) was added dropwise into a stirred solution of the above product and Cl_3_CCN (8 mL) in CH_2_Cl_2_ at 0 °C. The mixture was stirred for 2 h and concentrated. The residue was purified by column chromatography (petroleum ether–ethyl acetate = 3:1) to give intermediate **6** (3.45 g).

BF_3·_Et_2_O (1.5 mL, 15.3 mmol) was added dropwise into a stirred mixture of WS070117 (3.64 g, 7.50 mmol), intermediate **6** (5.4 g, 11.3 mmol) and 4 Å molecular sieves in CH_2_Cl_2_ (200 mL) at 0 °C. The mixture was stirred for 12 h and neutralized with 2 mL Et_3_N. The solution was concentrated *in vacuo* after filtration and the residue was purified by column chromatography (CH_2_Cl_2_–MeOH = 100:1) to give intermediate **7** (5.2 g, 86.7%). ESI/MS:802.53 [M + 1]^+^, ^1^H-NMR (300 MHz, DMSO-*d*_6_, see Supplementary Materials) δ: 10.07 (s, 1H), 8.56 (s, 1H), 8.44 (s, 1H), 7.80 (s, 1H), 7.65 (d, *J* = 8.2 Hz, 1H), 7.28 (t, *J* = 8.2 Hz, 1H), 6.71 (d, *J* = 9.8 Hz, 1H), 6.29 (d, *J* = 5.2 Hz, 1H), 6.06 (t, *J* = 5.6 Hz, 1H), 5.66 (dd, *J* = 10.7 Hz, 6.7 Hz, 2H), 5.52 (t, *J* = 9.6 Hz, 1H), 5.09 (m, 2H), 4.71 (d, *J* = 9.9 Hz, 1H), 4.43 (m, 2H), 4.28 (m, 1H), 3.64 (s, 3H), 2.13 (s, 3H), 2.03 (m, 15H).

### 3.8. Synthesis of N_6_-(3-O-β-d-Glucuronyphenyl) Adenosine *(**M4**)*

Cs_2_CO_3_ (2.5 g, 7.7 mmol) was added into a stirred solution of intermediate **7** (4.5 g, 5.6 mmol) in methanol (20 mL). The mixture was stirred at 40 °C for 0.5 h and then cooled to room temperature. The precipitated solid was filtered, redissolved with water (2 mL), and was then cation exchange resin was added to adjust the pH to 6–7. The solution was concentrated *in vacuo* after filtration to give **M4** (2.5 g, 83.3%) as a white solid, ${\left[\alpha \right]}_{D}^{20}$ = −51.2 (*c* = 0.1, H_2_O), ESI/MS:536.40 [M + H]^+^, ^1^H-NMR (400 MHz, methanol-*d*_4_, see Supplementary Materials) δ 8.42 (s, 1H), 8.38 (s, 1H), 7.95 (s, 1H), 7.37 (d, *J* = 8.2 Hz, 1H), 7.28 (t, *J* = 8.3 Hz, 1H), 6.84 (d, *J* = 8.2 Hz, 1H), 6.01 (d, *J* = 6.0 Hz, 1H), 5.01 (d, *J* = 6.3 Hz, 1H), 4.77 (t, *J* = 5.7 Hz, 1H), 4.34 (s, 1H), 4.18 (s, 1H), 4.02 (d, *J* = 10.0 Hz, 1H), 3.90 (d, *J* = 12.6 Hz, 1H), 3.76 (d, *J* = 12.5 Hz, 1H), 3.62 (d, *J* = 9.5 Hz, 1H), 3.53 (m, 2H). ^13^C-NMR (100 MHz, methanol-*d*_4_) δ 170.9, 158.0, 152.4, 151.8, 148.6, 140.9, 140.1, 129.1, 120.7, 114.2, 111.5, 108.8, 101.1, 89.8, 86.7, 76.0, 75.3, 74.1, 73.1, 71.7, 71.2, 62.0.

### 3.9. Synthesis of N_6_-(3-O-β-d-Glucuronyphenyl) Adenine *(**M2**)*

Concentrated hydrochloric acid (1 mL) was added into a stirred solution of **M4** (1.6 g, 3 mmol) in water (10 mL). The reaction mixture was refluxed for 1 h and then cooled to room temperature. The precipitated solid was filtered and washed with water to give **M2** (0.89 g, 74.1%) as a white solid, ${\left[\alpha \right]}_{D}^{20}$ = −139.8 (*c* = 0.08, H_2_O), ESI/MS: 404.33 [M + H]^+^, ^1^H-NMR (400 MHz, DMSO-*d*_6_, see Supplementary Materials) δ 9.95 (brs, 1H), 8.42 (s, 1H), 8.27 (s, 1H), 7.89 (s, 1H), 7.65 (d, *J* = 7.2 Hz, 1H), 7.23 (t, *J* = 8.1 Hz, 1H), 6.70 (d, *J* = 7.9 Hz, 1H), 5.00 (d, *J* = 7.1 Hz, 1H), 3.88 (d, *J* = 9.0 Hz, 1H), 3.34 (m, 3H). ^13^C-NMR (100 MHz, DMSO-*d*_6_) δ 170.6, 157.7, 152.0, 151.5, 141.4, 141.1, 129.6, 114.4, 110.1, 108.7, 100.5, 76.4, 76.1, 73.4, 71.9.

## 4. Conclusions

Seven metabolites of 2′,3′,5′-tri-*O*-acetyl-*N*_6_-(3-hydroxyphenyl) adenosine (WS070117) were successfully synthesized by deacetylation, hydrolysis, cyclization, sulfonylation and glycosylation reactions. All these compounds were characterized by NMR and HPLC-MS (MS) analyses, and the purities (HPLC) are all higher than 98%, so they could be used as material standards for metabolic research on WS070117.

## Supplementary Materials

Supplementary materials can be accessed at: http://www.mdpi.com/1420-3049/21/1/8/s1.
