# Supplementary material for: The Synthesis of the Metabolites of 2′,3′,5′-Tri-O-acetyl-N6-(3-hydroxyphenyl) Adenosine (WS070117)
Source: Molecules. 2015 Dec 28;21(1):8. doi: 10.3390/molecules21010008 (PMC6273794; doi:10.3390/molecules21010008)
Supplement: Supplementary file 1 [file molecules-21-00008-s001.pdf]

Supplementary Materials: The Synthesis of the Metabolites of 2′,3′,5′-Tri-*O*-acetyl-*N*6-(3-hydroxyphenyl) Adenosine (WS070117)

Wen-Xuan Zhang, Hong-Na Wu, Bo Li, Hong-Lin Wu, Dong-Mei Wang and Song Wu *

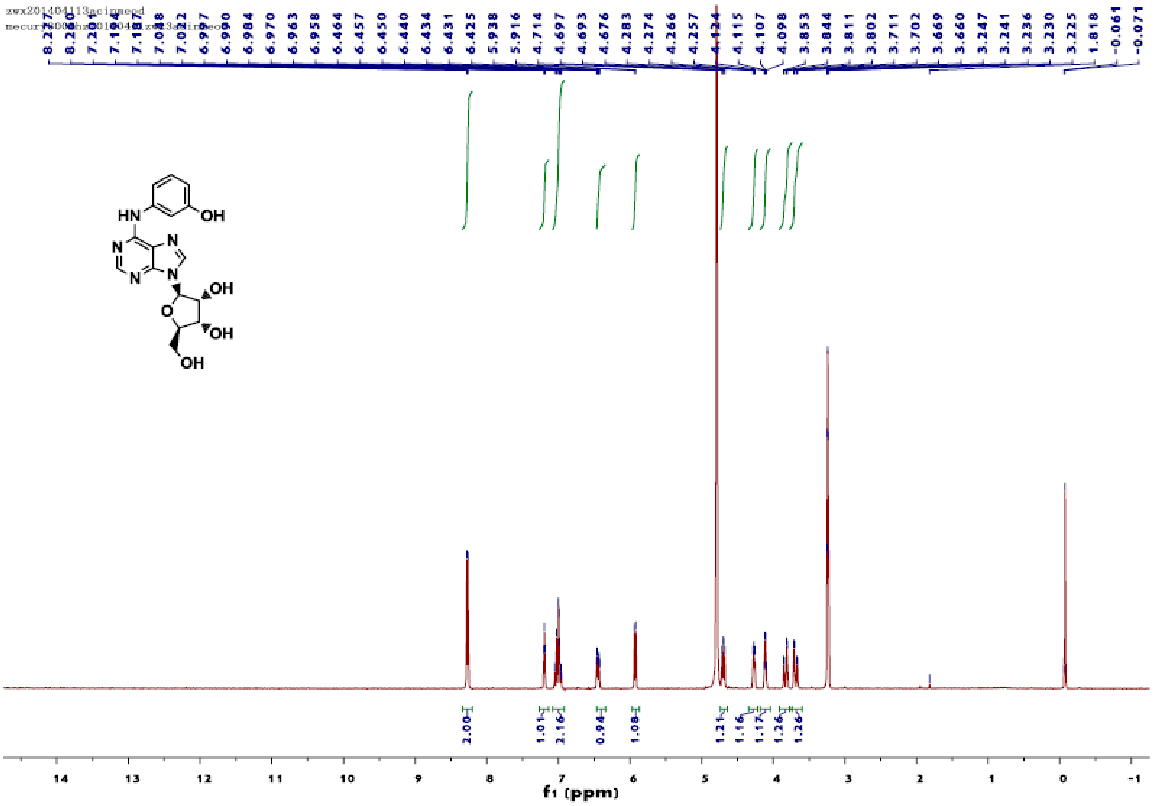

Figure S1. The ¹H-NMR of metabolites of **M8**.

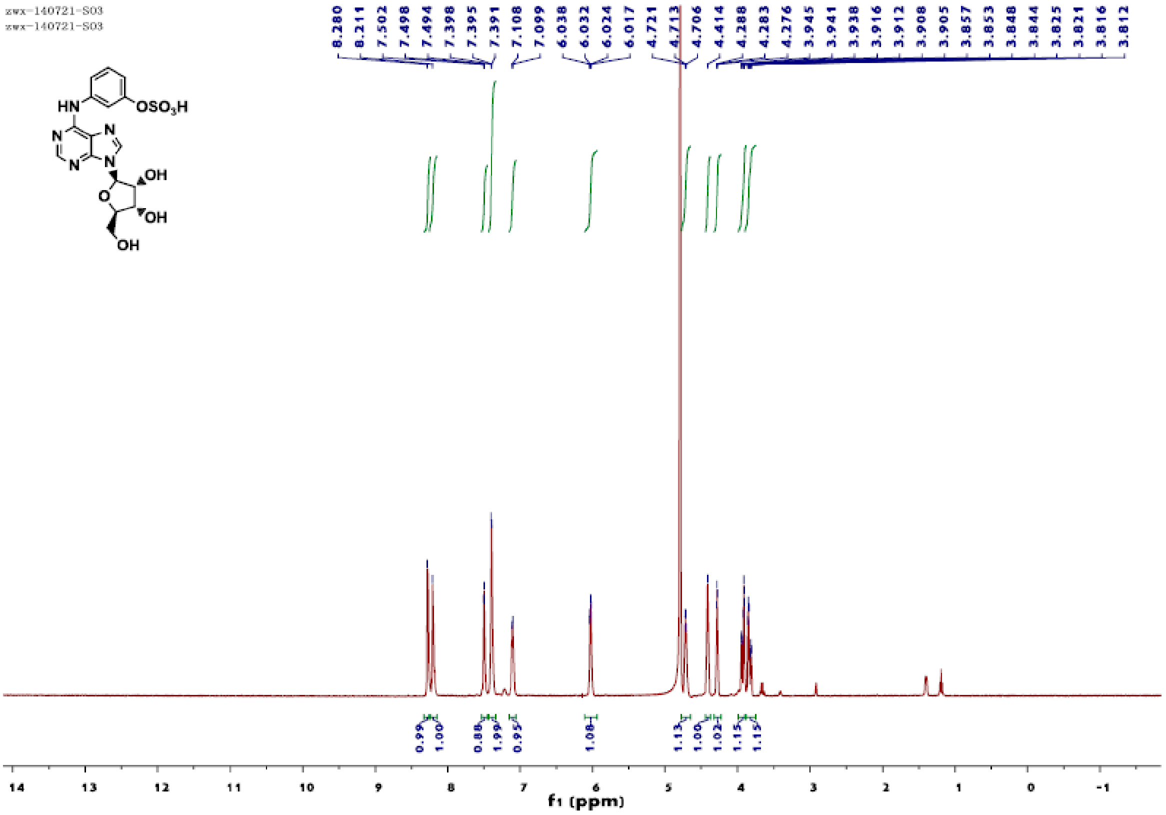

Figure S2. The ^{1}H-NMR of metabolites of **M5**.

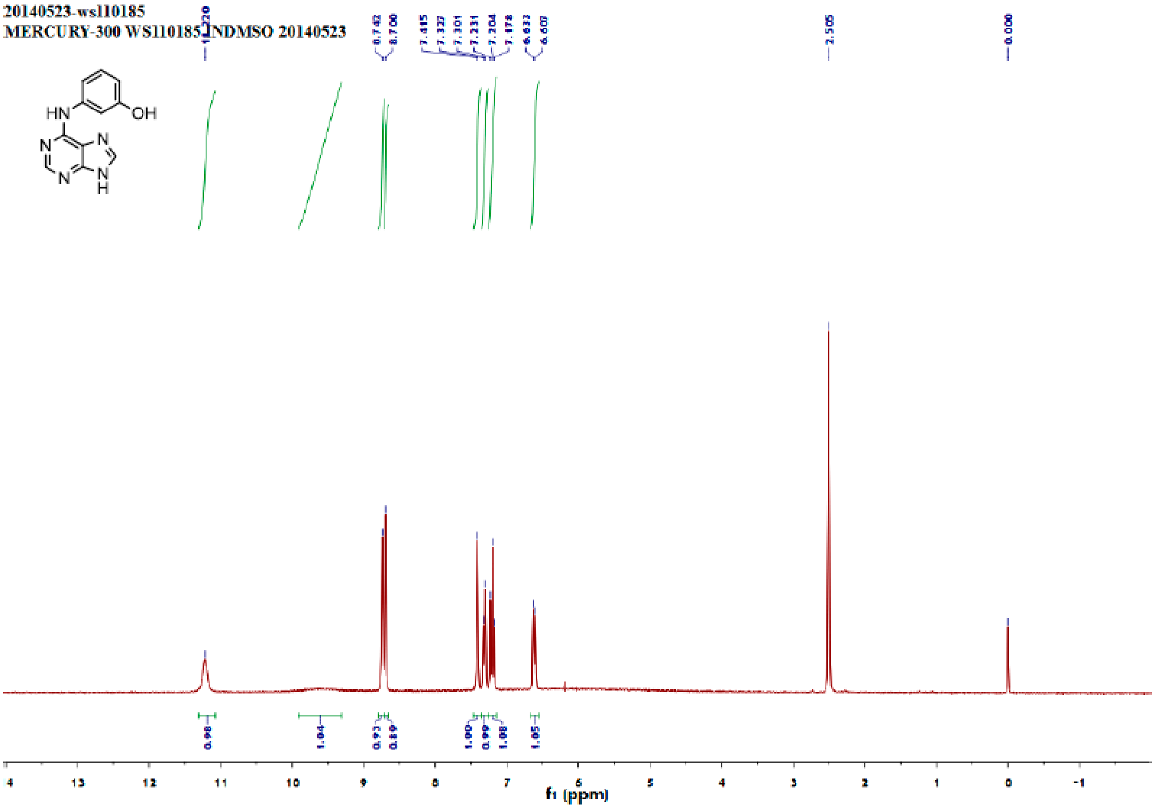

Figure S3. The ^{1}H-NMR of metabolites of **M7**.

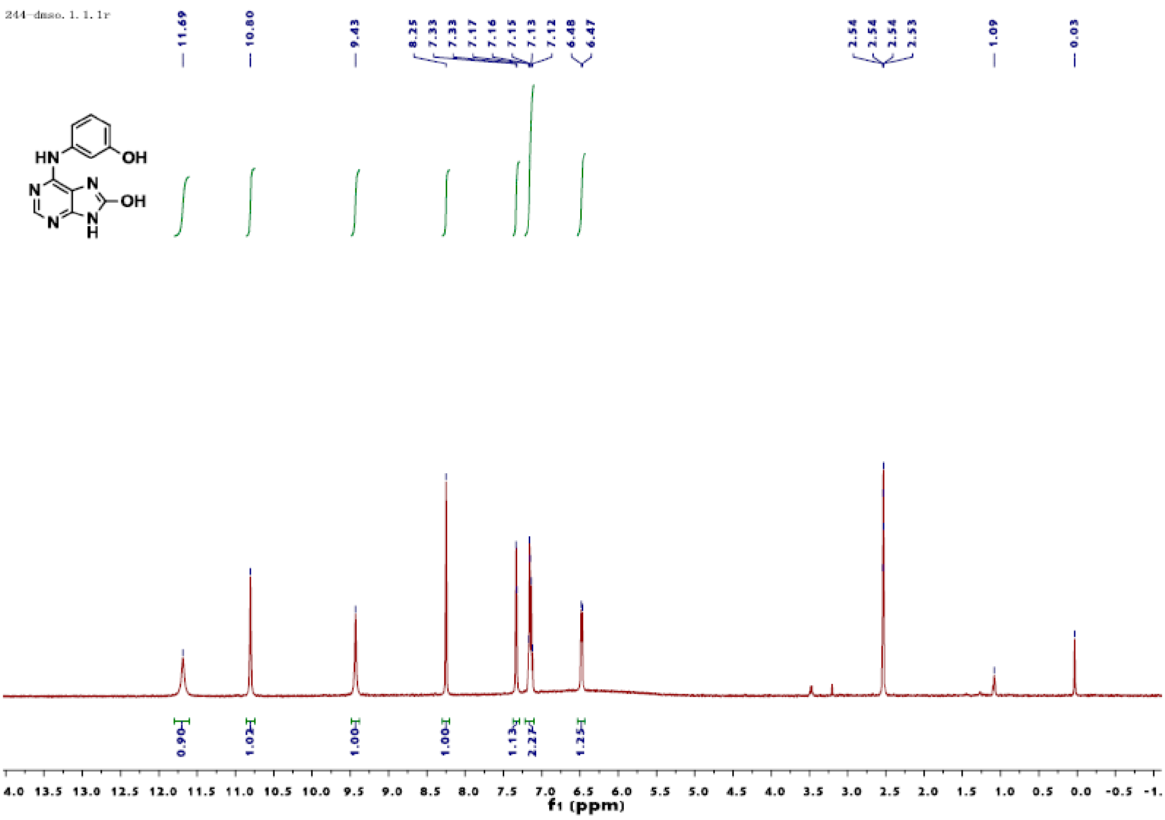

Figure S4. The ¹H-NMR of metabolites of **M6**.

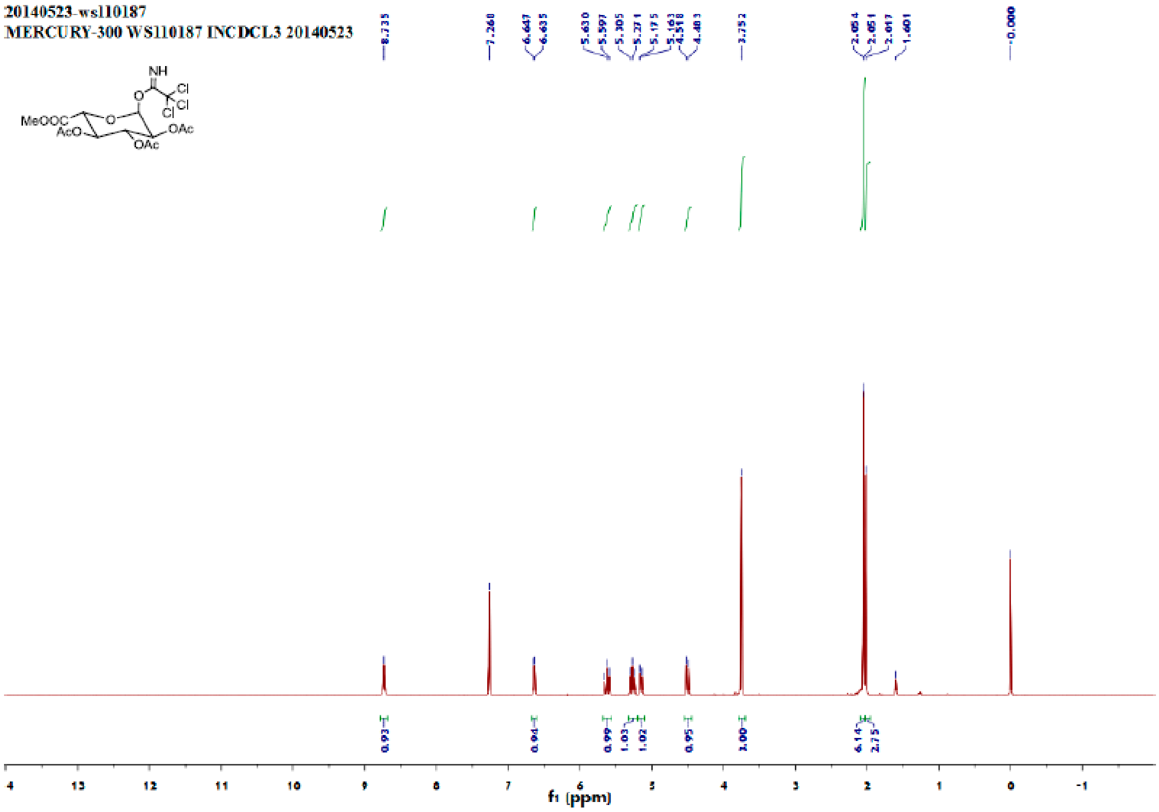

Figure S5. The ¹H-NMR of intermediate of **6**.

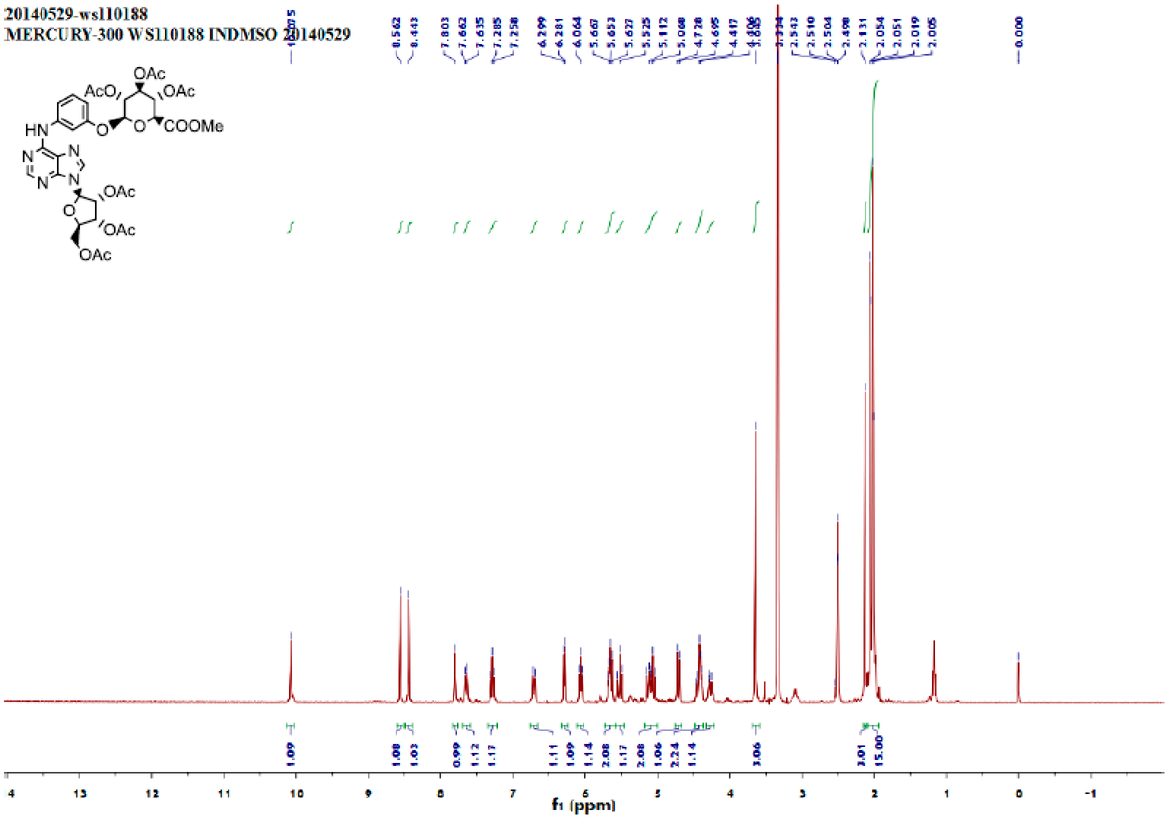

Figure S6. The ^{1}H-NMR of intermediate of **7**.

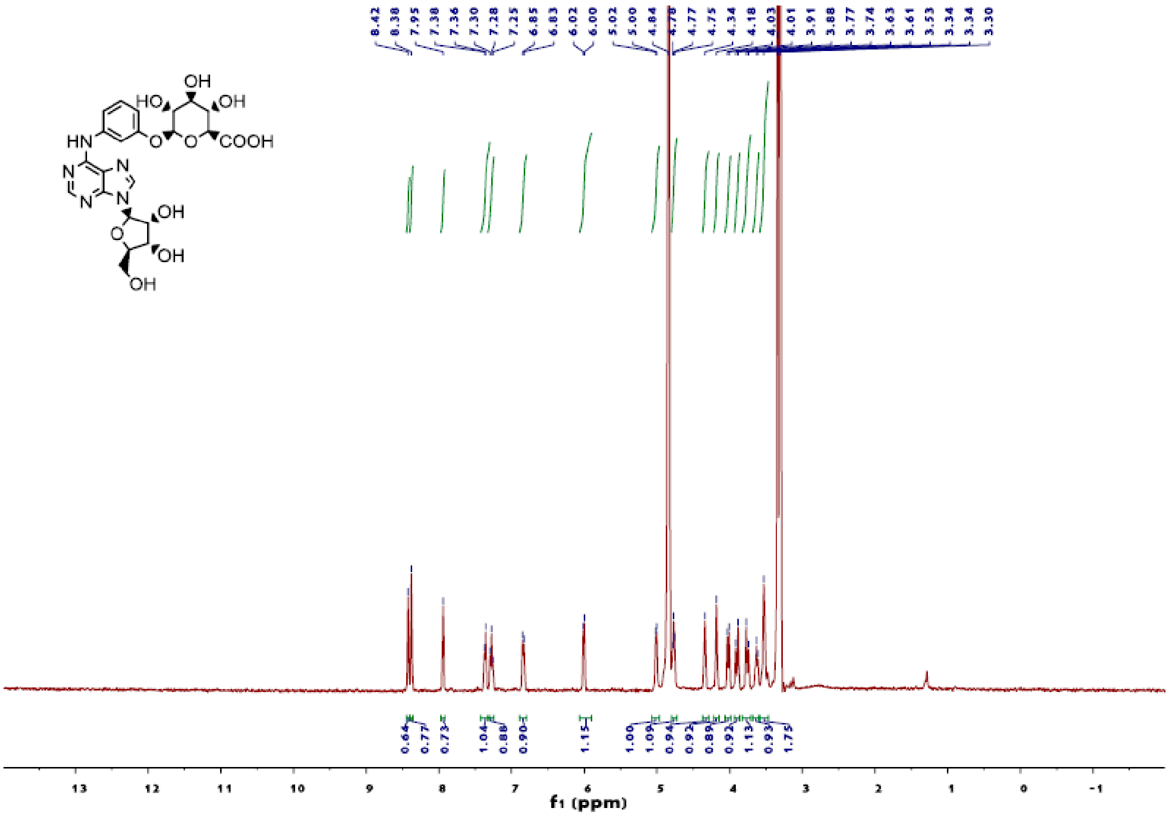

Figure S7. The ^{1}H-NMR of metabolites of **M4**.

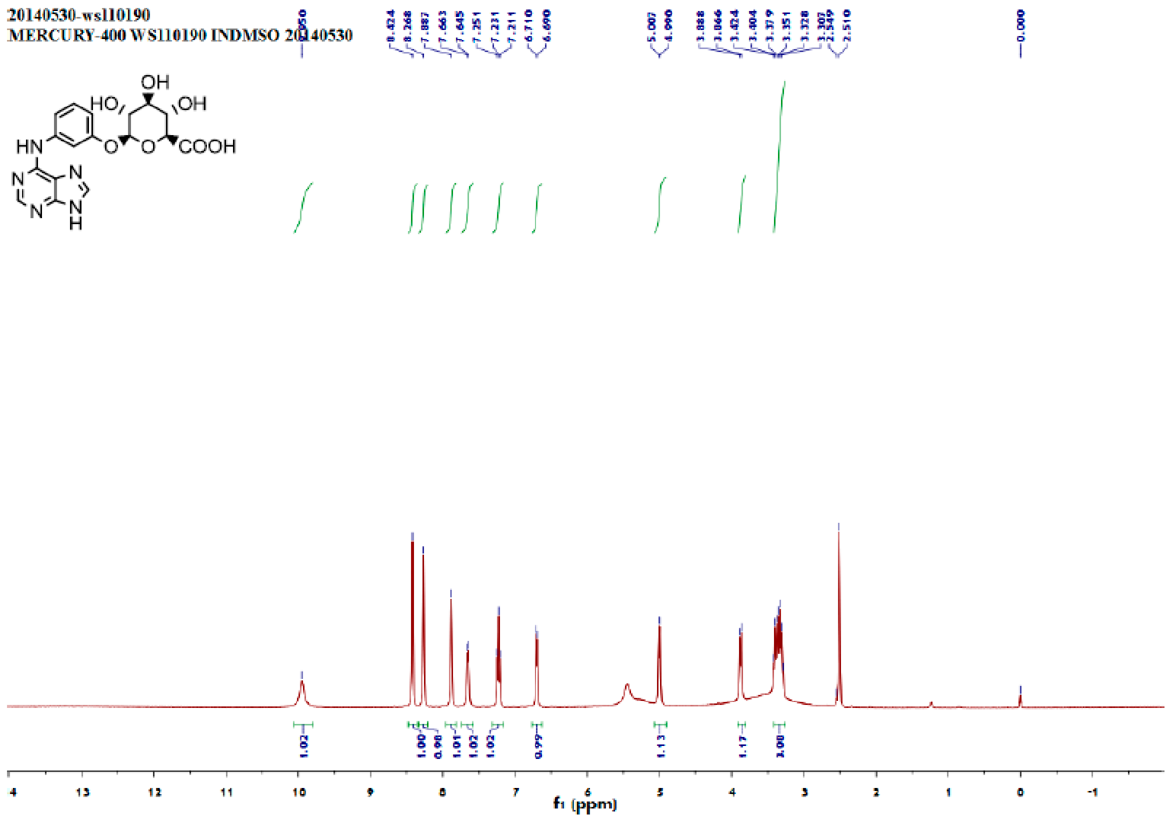

Figure S8. The ^{1}H-NMR of metabolites of **M2**.
